# Timing and co-occurrence of symptoms prior to a diagnosis of light chain (AL) amyloidosis

**DOI:** 10.21203/rs.3.rs-3788661/v1

**Published:** 2024-01-03

**Authors:** Anita D’Souza, Ashima Singh, Aniko Szabo, Qinghua Lian, Liliana Pezzin, Rodney Sparapani

**Affiliations:** Medical College of Wisconsin; Medical College of Wisconsin; Medical College of Wisconsin; Medical College of Wisconsin; Medical College of Wisconsin; Medical College of Wisconsin

**Keywords:** early diagnosis, precursor diagnoses, amyloid

## Abstract

It is well-established that light chain (AL) amyloidosis patients have multi-organ involvement and are often diagnosed after a lag period of increasing symptoms. We leverage electronic health record (EHR) data from the TriNetX research network to describe the incidence, timing, and co-occurrence of precursor conditions of interests in a cohort of AL amyloidosis patients identified between October 2015-December 2020. Nineteen precursor diagnoses of interest representing features of AL amyloidosis were identified using ICD codes up to 36 months prior to AL amyloidosis diagnosis. Among 1,401 patients with at least 36 months of EHR data prior to AL amyloidosis diagnosis, 46% were females, 16% were non-Hispanic Black, and 6% were Hispanic. The median age was 71 (range, 21–91) years. The median number of precursor diagnoses was 5 with dyspnea and fatigue being the most prevalent. The time from the first occurrence of a precursor to AL diagnosis ranged from 3.2 to 21.4 months. Analyses of pairwise co-occurrence of specific diagnoses indicated a high association (Cole’s coefficient > 0.6) among the examined precursor diagnoses. These findings provide novel information about the timing and co-occurrence of key precursor conditions and could be used to develop algorithms for early identification of AL amyloidosis.

## Background

Light chain (AL) amyloidosis is a rare plasma cell disorder characterized by extracellular tissue deposition of misfolded and aggregated amyloid fibrils derived from clonal immunoglobulin-free light chains.(1) The majority of patients with AL amyloidosis present with multisystemic involvement, where heart, kidneys, gastrointestinal tract, nervous system, and musculoskeletal system often affected.(2) The disease often entails distinct and disparate symptoms across different organ systems over time leading to healthcare visits with multiple specialists.(3) Existing literature suggests that more than one-third of patients report symptoms for a year or longer and approximately half sought four or more different physicians before their AL amyloidosis diagnosis was formally established.(3) Literature and clinical experience suggests a substantial delay in diagnosis from initial onset of symptoms.(3–7) The delay in AL amyloidosis diagnosis leads to many patients being diagnosed with advanced organ involvement, often associated with poor prognosis.(1)

Reducing the time from the onset of precursor conditions’ symptom to the diagnosis for AL amyloidosis is a critical unmet need. Taken together, the low incidence of AL amyloidosis, the non-specificity of its presenting symptoms, and the resulting reliance on numerous different healthcare providers to address them make disease diagnosis a complex task. For these reasons, disease awareness and a high suspicion by the provider physician are key elements to making a diagnosis of AL amyloidosis. In this paper, we contribute toward that goal by examining the incidence, timing, and co-occurrence specific conditions likely to be symptoms related to AL amyloidosis (we refer them as precursor diagnoses). Specifically, we leverage a large and diverse electronic health record (EHR) dataset to describe the timing of nineteen specific clinical precursor diagnoses and their co-occurrence within the three years prior to the patients’ diagnosis of AL amyloidosis.

## Methods

### Data source

Data for this observational retrospective cohort study of patients diagnosed with AL amyloidosis were drawn from TriNetX. TriNetX is a health research network providing access to high-quality, de-identified patient-level EHR data from more than 60 U.S. healthcare organizations. These data include diagnoses, visits, prescriptions, procedures ordered, vital signs, laboratory values, and are refreshed on a regular basis.

#### Cohort identification:

As a first step, we selected a cohort of individuals with AL amyloidosis by identifying patients who had at least one inpatient or at least two outpatient visits with an association AL amyloidosis International Classification of Diseases (ICD) diagnosis code (ICD-9: 277.30, 277.39 or ICD-10: E85.81, E85.4, E85.89, E85.9) during the study period between 10/01/2015–12/31/2020. The date of the earliest of these occurrences was used to indicate the time of the patients’ formal AL amyloidosis diagnosis. In order to make the cohort specific to individuals with AL amyloidosis who might have been picked by the amyloidosis unspecified ICD9 code E85.9, we further required patients to have received chemotherapy or autologous BMT within − 90 to + 365 days of the AL amyloidosis diagnosis for the study. We restricted the sample to individuals for whom there was information on healthcare utilization in TriNetX dating back to at least 3 years from their AL amyloidosis diagnosis date. Our choice of look back period was consistent with the prior literature indicating that nearly 90% of AL amyloidosis patients report initial symptoms within 3 years and 80% within 2 years of their AL amyloidosis diagnosis(3).

#### Variable Definitions.

The precursor conditions of interest, along with their ICD codes, are listed in Table 1. These were categorized by organ system as clonal, cardiac, renal, gastrointestinal, multisystemic, and neurologic, with a residual category of miscellaneous. Time from precursor condition to AL diagnosis was calculated based on the date of the earliest medical encounter with a code for the specific precursor diagnosis to the date of AL amyloidosis diagnosis.

### Statistical analysis

The onset of AL amyloidosis diagnosis was considered as time 0 and the EHR period preceding time 0 was shown 6 months prior, 12 months prior, 24 months prior, and 36 months prior to time 0. By cohort definition, all patients had a minimum of 36 months of EHR data prior to time 0.

The first period, 36 months to 24 months prior to time 0 was considered as the prevalence period to determine the baseline prevalence of the precursor diagnoses within the cohort assuming that it may not be due to AL amyloidosis. Starting 24 months prior to time 0, the new appearance of precursor diagnoses was considered as the incidence. For each precursor diagnosis, the incidence was calculated by determining the proportion of patients who had a new occurrence of the specific precursor diagnosis code starting 24 months prior to the AL amyloidosis diagnosis code to the first occurrence of AL diagnosis. The median time between the first instance of each precursor diagnosis to time 0 was estimated via a kernel-density estimation and using a scaled probit transformation to account for the boundary restrictions at −36 months and time 0.

The pairwise co-occurrence between precursor diagnoses was calculated using the Cole’s coefficient (CC). This coefficient measures the degree to which the observed proportion of joint occurrences exceeds or falls short of the proportion of joint occurrences expected by chance alone.(9) It equals 0 when there is no association between the events, achieves the value of + 1 when one event is a subset of the other. Given the exploratory nature of our analysis, correlations greater than 0.6 were considered strong. Fisher’s exact test was used to assess the statistical significance of the co-occurrence.

## Results

There were 1,401 patients with AL amyloidosis with at least 3 years of EHR history available. The median age of the cohort was 71 (range 21–91) years, with 646 (46%) of the patients being females, 877 (63%) non-Hispanic White, 79 (5.6%) Hispanic, 225 (16%) non-Hispanic Black, 17 (1.2%) non-Hispanic other, and 203 (14%) unknown race and/or ethnicity by self-report. The median length of time with medical history available in EHR was 5.7 years (range, 3.0–10 years). A median of 4 [Interquartile range (IQR) = 2–5] organ systems were identified among precursor diagnoses codes prior to AL amyloidosis diagnosis, with a median of 5 (IQR = 2–9) specific precursor diagnoses. The number of diagnosed precursor conditions increased from an average of two at 24 months prior to the AL amyloidosis diagnosis to three at 12 months to four at six months prior to the identification of AL amyloidosis, Table 2.

The most prevalent precursor diagnoses observed as a diagnostic code at AL amyloidosis diagnosis were as follows; dyspnea (55.3%), fatigue (44.7%), neuropathy (39.9%), chronic kidney disease (37.5%), altered bowels (37.0%), edema (36.5%), and heart failure (36.3%). In contrast, less than 3% of the patients had an incidence of a diagnostic code for purpura (2.4%), nephritic syndrome (2.3%), nephrotic syndrome (2.3%), autonomic neuropathy (0.9%), and macroglossia (0.2%), Table 3. The median time to AL amyloidosis diagnosis from the earliest precursor condition ranged from 3.2–21.4 months.

**Figure 1:**
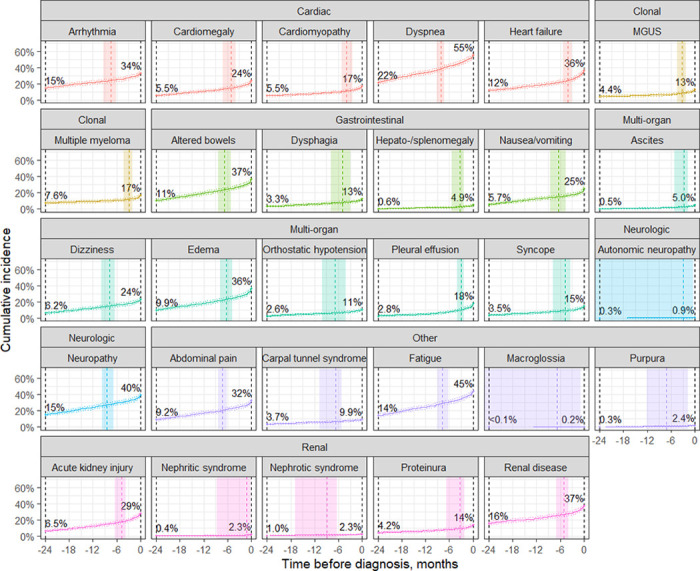
Development of precursor diagnoses prior to AL amyloidosis diagnosis. Cumulative incidence of diagnoses among patients with 3+ years EHR history during the incidence period, with dotted lines showing pointwise 95% confidence limits. The dashed vertical line shows the median diagnosis time during this period with 95% confidence interval shown by shading. The percentage of patients with a diagnosis by the start and end of the incidence period as shown.

As illustrated in Fig. 1, the incidence of precursor conditions within 2 years of AL amyloidosis diagnosis varied between 0.14% (macroglossia) to 34% (dyspnea). The estimated probability density of each precursor diagnosis onset by time before AL amyloidosis diagnosis are provided in the supplemental figures showing the density in the entire 24 month prior (Suppl Fig. 1) and preceding 6 months prior to AL amyloidosis diagnosis when the appearance of new precursor diagnosis is the greatest (Suppl Fig. 2).

**Figure 2:**
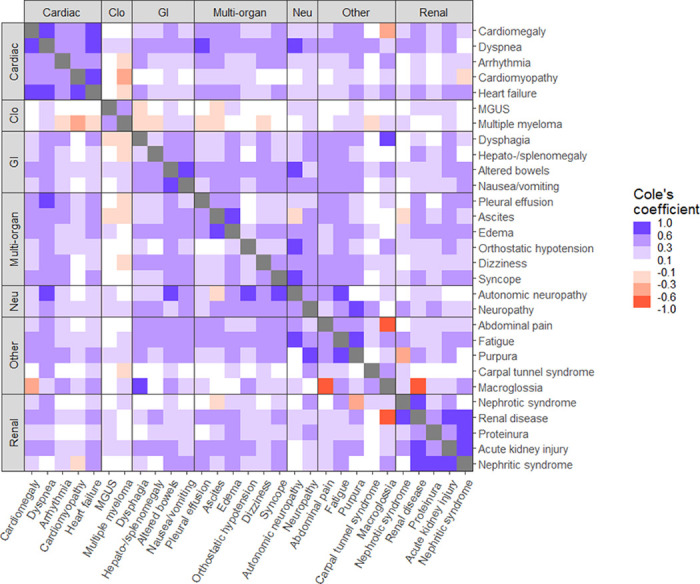
Co-occurrence of precursor diagnoses The extent of co-occurrence of diagnoses was quantified using Cole’s coefficient. It equals 0 when there is no association between the events, achieves the value of +1 when one event is a subset of the other, and a value of −1 if the events never co-occur. Fisher’s exact test was used to assess the statistical significance of the co-occurrence.

In terms of co-occurrence of symptoms, the strongest positive correlations were observed for the co-occurrence of dysphagia and macroglossia (CC = 0.86, p-value = 0.002), autonomic neuropathy and dyspnea (CC = 0.76, p-value = 0.009), heart failure and cardiomyopathy (CC = 0.74, p-value < 0.001), renal disease and nephrotic syndrome (CC = 0.74, p-value < 0.001). Other interesting associations included purpura and neuropathy (CC = 0.68, p-value < 0.001), fatigue and autonomic neuropathy (CC = 0.68, p-value = 0.004), purpura and fatigue (CC = 0.65, p-value < 0.001), syncope and autonomic neuropathy (CC = 0.62, p-value < 0.001), and altered bowels and autonomic neuropathy (CC = 0.60, p 0.006). Table 4 lists all precursor diagnoses and organ systems co-occurring with significant and strong correlation co-efficient. Figure 2 shows the pairwise associations among all the tested precursor diagnoses.

## Discussion

The diagnosis of AL amyloidosis is often delayed despite patients reporting multiple symptoms and seeing different specialists for care over several months to years. This context underscores the critical unmet need for reducing the time from the initial onset of symptoms to the diagnosis of the disease. To address this need, we leveraged a large EHR dataset to investigate the timing and co-occurrence of specific precursor diagnoses occurring before the diagnosis of AL amyloidosis. We were particularly interested to understand when and how certain precursor diagnoses such as dyspnea, fatigue, edema, pain, proteinuria, among others were established as diagnoses within the medical history as a diagnostic code in relation to the AL amyloidosis diagnosis. These precursor diagnoses were derived from symptoms and signs endorsed by many AL amyloidosis patients.(4, 10) We were interested in understanding whether these get catalogued as diagnoses within medical history by ICD codes and if so, how early before the diagnosis of AL amyloidosis. By identifying the proportion of patients with these precursor diagnoses and examining their timing and co-occurrence, our study sheds light on the diagnostic process using EHR data in this rare multisystemic condition.

Our prior work suggests that AL amyloidosis patients have a high prevalence of precursor diagnoses.(6) In the current analyses, we studied the pattern and timing of these diagnoses prior to the diagnosis of AL amyloidosis using the same data source. Concordant with findings by others (7) and as reported in patient surveys,(3) our analysis confirms the high prevalence of several symptoms of the disease present and diagnosed in EHR seen in this disease well before the diagnosis of AL amyloidosis has been made with a median time of 3.2 to 21.4 months before AL amyloidosis diagnosis, providing support to efforts to develop predictive algorithms toward early diagnosis.

The most common precursor diagnoses include dyspnea, fatigue, heart failure, edema, altered bowels, neuropathy, and chronic kidney disease. Fatigue is the most common symptom of the disease as reported by 80% of AL amyloidosis patients.(4) Our data show that fatigue is also the most catalogued of the precursor diagnoses as an ICD code, seen in 45% of patients with median time 15.6 months before the diagnosis of AL amyloidosis. Other common AL amyloidosis symptoms including dyspnea and edema are also commonly identified as ICD codes by healthcare providers at one year or longer prior to the diagnosis of AL amyloidosis. This concordance with known symptoms of the disease document the feasibility to using EHR data of diagnosis codes toward creating algorithms that could improve time from symptom onset to AL amyloidosis diagnosis.

When assessing co-occurrence of precursor diagnoses, the strongest correlation was often seen with precursor diagnoses within the same organ system/category, for e.g., cardiomyopathy and heart failure, or nephrotic syndrome and renal disease. This was concordant with expected AL amyloidosis pathology, in that, with organ involvement, the disease would be expected to cause multiple symptoms and signs related to that organ system. Other precursor diagnoses belonged to different organ systems/categories but made intuitive sense as a downstream effect of one of the precursors e.g., autonomic neuropathy and syncope or macroglossia and dysphagia.

The majority of AL amyloidosis patients have more than one organ involvement, thus correlations between organ systems was of greater interest in our analysis. Here we saw numerous strong correlations across various organ system categories, autonomic neuropathy and dyspnea, neuropathy and purpura, fatigue and purpura, fatigue and autonomic neuropathy. The two organ systems which showed the greatest correlation included cardiac and gastrointestinal, cardiac and multisystemic, gastrointestinal and other, neurologic and multisystemic, and neurologic with other.

It is crucial to acknowledge and consider the limitations of our study, inherent to the use of EHR data in research, when interpreting the results. For example, misdiagnoses, coding errors, and variations in which symptoms are recorded can lead to incorrect associations or missed precursor diagnoses. Different healthcare organizations within the TriNetX network may have variations in EHR systems and diagnostic coding practices, thus limiting the generalizability of the findings. The early symptoms of AL amyloidosis are nonspecific and can mimic other, more common, conditions. Our study assumes that precursor diagnoses represent early symptoms of AL amyloidosis, but we have not adjusted for comorbidities that may drive the onset of many of the precursor diagnoses. For example, presence of diabetes may lead to neuropathy, proteinuria, and cardiomyopathy. Our approach in selecting a cohort with at least 3 years of backward medical history allows us to assess a baseline prevalence period in the first year of EHR history and then incidence in the subsequent two years preceding the AL amyloidosis diagnosis. Lastly, we lack detailed clinical context making it challenging to understand the severity and clinical significance of precursor diagnoses. Nevertheless, our approach is relevant because it is reflective of symptoms that are clinically recognized by healthcare providers. A big strength of our analysis is the racial diversity of our dataset often lacking in published clinical research in AL amyloidosis from the U.S.(11)

In conclusion, leveraging real time EHR data enabled us to identify a large and diverse cohort of AL amyloidosis patients from which to examine diagnostic patterns and demonstrate the potential for earlier diagnosis of this complex disease. Our findings lay the foundation to develop clinical algorithms using ICD codes aimed at earlier recognition of AL amyloidosis.

## Figures and Tables

**Table 1.** ICD codes for the precursor diagnoses of interest

Diagnoses	ICD 9	ICD 10

**Clonal**		

MGUS	D47.2	273.1

Multiple myeloma	203	C90

**Cardiac**		

Cardiomegaly	429.3	I51.7

Cardiomyopathy	425.4	I42.5
	425.18	I42.2
	425.8	I43*

Heart failure	428.3	I50.3
	428.9	I50.9
	428	I50.8

Arrhythmia	427.9	I49.9
	427.89	I49.8
	427.3	I48*
	427.31	

Dyspnea	786*	R06.0*

**Multisystemic**		

Edema	782.3	R60
		R60.1
		R60.9

Pleural effusion	511*	J90*
		J91*

Ascites	789.5*	R18*

Syncope	780.2	R55

Orthostatic hypotension	458	I95.1

Dizziness	780.4	R42

**Renal**		

Acute kidney injury	584.9	N17.9
		S37.0

Nephritic	583.9	N05

Nephrotic	583.81	N08

Proteinura	791	R80.1
		R80.9
		R80.8

Chronic kidney disease	585*	N18*

**Gastrointestinal**		

Dysphagia	787.2*	R13.1*

Hepatomegaly and/or splenomegaly	789.1	R16.0
	789.2	R16.1
		R16.2

Altered bowels	787.99	R19.4
	787.91	R19.7
	558.9	K52.9
	564.1	K58.0
	564.5	K59.1
	564	K59.0

Nausea and/or vomiting	787.01	R11.0
	787.02	R11.10
	787.03	R11.2

**Neurologic**		

Neuropathy	787.01	R11.2
	782	R20.2
	729.2	R20.9
	357.89	M79.2
	356.4	G64
	357.9	G60.3
		G62.9
		G62.8

Autonomic neuropathy	337.9	G90.0
	337.1	G90.9
	337.09	G99.0
		G90.09

**Other**		

Macroglossia	750.15	Q38.2

Fatigue	780.7*	R53*

Purpura	782.7	R23.3
	287.8	D69.8
	287.9	D69.9

Abdominal pain	789*	R10*

Carpal tunnel syndrome	354*	G56*

**Table 2:** Cumulative number of precursor diagnoses over time prior to AL amyloidosis diagnosis

Characteristic	−24 months pre-AL amyloidosis	−6 months per-AL amyloidosis	At AL amyloidosis diagnosis
Number of precursor diagnoses			
Mean (SD)	2.9 (3.3)	4.8 (4.1)	6.0 (4.6)
Median (IQR)	2 (0–4)	4 (2–7)	5 (2–9)
Number of organ systems			
Mean (SD)	1.9 (1.7)	3.0 (1.9)	3.4 (2.0)
Median (IQR)	2 (0–3)	3 (1–5)	4 (2–5)

SD- standard deviation, IQR- inter-quartile range

**Table 3:** Proportion of patients with precursor diagnoses based on time before AL amyloidosis diagnosis

Precursor diagnosis	Incidence within 2 years pre-AL amyloidosis (95% CI)	Prevalence at AL amyloidosis diagnosis (95% CI)	Median time to precursor before AL diagnosis, months (IQR)
Clonal			
MGUS	9% (17–21%)	13% (11–15%)	3.2 (0.6, 8.2)
Smoldering multiple myeloma	9% (8–11%)	17% (15–19%)	2.9 (0.5, 10.0)
Cardiac			
Dyspnea	34% (31–36%)	55% (53–58%)	8.1 (2.7, 15.9)
Heart failure	24% (22–26%)	36% (34–39%)	4.1 (1.2, 11.7)
Arrhythmia	19% (17–21%)	34% (31–36%)	7.5 (1.8, 16.7)
Cardiomegaly	19% (17–21%)	24% (22–26%)	5.2 (0.9, 13.7)
Cardiomyopathy	12% (10–13%)	17% (15–19%)	3.9 (1.0, 11.2)
Renal			
Acute kidney injury	22% (20–24%)	29% (26–31%)	4.8 (1.3, 12.6)
Renal disease	22% (20–24%)	38% (35–40%)	5.2 (1.2, 12.7)
Proteinuria	10% (9–12%)	14% (12–16%)	3.3 (0.8, 10.8)
Nephritic syndrome	2% (1–3%)	2% (1.5–3%)	1.2 (0.1, 8.6)
Nephrotic syndrome	1% (0.7–2%)	2% (1.5–3%)	8.8 (4.2, 15.8)
Gastrointestinal			
Altered bowels	26% (24–29%)	37% (35–40%)	6.7 (1.5, 14.9)
Abdominal pain	23% (21–26%)	33% (30–35%)	7.4 (1.4, 15.4)
Nausea and/or vomiting	19% (17–21%)	25% (23–27%)	6.4 (1.1, 15.8)
Dysphagia	10% (8–11%)	13% (11–15%)	5.0 (0.8, 13.9)
Hepatomegaly and/or splenomegaly	4% (3–5%)	5% (4–6%)	3.3 (0.7, 11.9)
Neurologic			
Neuropathy	25% (22–27%)	40% (37–43%)	8.5 (1.8, 15.7)
Autonomic neuropathy	1 % (0.2–1 %)	1% (0.4–1.4%)	3.0 (0.4, 12.9)
Multisystemic			
Edema	27% (24–29%)	37% (34–39%)	6.3 (1.2, 14.9)
Dizziness	17% (15–19%)	24% (21–26%)	7.8 (1.7, 15.5)
Pleural effusion	15% (13–17%)	1% (16–20%)	2.9 (0.6, 8.1)
Syncope	11% (10–13%)	15% (13–17%)	4.7 (1.0, 13.0)
Orthostatic hypotension	8% (7–9%)	11 % (9–39%)	6.9 (1.9, 16.9)
Ascites	5% (3–6%)	5% (4–6%)	2.7 (0.5, 8.4)
Other			
Fatigue	30% (28–33%)	45% (42–47%)	7.8 (1.9, 15.8)
Carpal tunnel syndrome	6% (5–7%)	10% (8–35%)	6.7 (1.8, 15.1)
Purpura	2% (1–3%)	2% (2–3%)	7.2 (0.8, 13.2)
Macroglossia	0.14% (NA, 0.3%)	0.2% (NA, 0.5%)	6.8 (3.8, 9.8)

**Table 4:** Cole’s co-efficient values for co-occurrence between precursor diagnoses

Organ system 1	Precursor diagnosis 1	Organ system 2	Precursor diagnosis 2	Cole’s Coefficient	p-value
Gastrointestinal	Dysphagia	Other	Macroglossia	0.86	0.002
Neurologic	Autonomic neuropathy	Cardiac	Dyspnea	0.76	0.009
Cardiac	Cardiomyopathy	Cardiac	Heart failure	0.74	< 0.001
Renal	Nephrotic syndrome	Renal	Renal disease	0.73	< 0.001
Renal	Nephritic syndrome	Renal	Renal disease	0.68	< 0.001
Neurologic	Neuropathy	Other	Purpura	0.68	< 0.001
Renal	Acute kidney injury	Renal	Nephritic syndrome	0.68	< 0.001
Cardiac	Cardiomegaly	Cardiac	Dyspnea	0.68	< 0.001
Neurologic	Autonomic neuropathy	Other	Fatigue	0.68	0.004
Cardiac	Dyspnea	Cardiac	Heart failure	0.67	< 0.001
Other	Fatigue	Other	Purpura	0.65	< 0.001
Cardiac	Dyspnea	Multi-organ	Pleural effusion	0.65	< 0.001
Renal	Acute kidney injury	Renal	Renal disease	0.65	< 0.001
Neurologic	Autonomic neuropathy	Multi-organ	Orthostatic hypotension	0.64	< 0.001
Renal	Nephritic syndrome	Renal	Proteinura	0.63	< 0.001
Neurologic	Autonomic neuropathy	Multi-organ	Syncope	0.62	< 0.001
Cardiac	Cardiomegaly	Cardiac	Heart failure	0.62	< 0.001
Multi-organ	Ascites	Multi-organ	Edema	0.61	< 0.001
